# A Comprehensive Review of Management Strategies for Bicuspid Aortic Valve (BAV): Exploring Epidemiology, Aetiology, Aortopathy, and Interventions in Light of Recent Guidelines

**DOI:** 10.3390/jcdd10090398

**Published:** 2023-09-18

**Authors:** Halil Ibrahim Bulut, Arian Arjomandi Rad, Angeliki-Aikaterini Syrengela, Iakovos Ttofi, Jasmina Djordjevic, Ramanjit Kaur, Amar Keiralla, George Krasopoulos

**Affiliations:** 1 Cerrahpasa School of Medicine, Istanbul University-Cerrahpasa, Istanbul 34098, Turkey; halilibrahim.bulut@ogr.iuc.edu.tr; 2 Medical Sciences Division, University of Oxford, Oxford OX1 3AZ, UK; 3 School of Medicine of Crete, 71500 Heraklion, Greece; aggsyr@yahoo.gr; 4 Department of Cardiothoracic Surgery, Oxford University Hospital NHS Foundation Trust, Oxford OX3 9DU, UK; iakovos.ttofi@ouh.nhs.uk (I.T.); jasmina.djordjevic@ouh.nhs.uk (J.D.); ramanjit.kaur@ouh.nhs.uk (R.K.); amar.keiralla@ouh.nhs.uk (A.K.)

**Keywords:** BAV, bicuspid aortic valve

## Abstract

Objective: bicuspid aortic valve (BAV) stands as the most prevalent congenital heart condition intricately linked to aortic pathologies encompassing aortic regurgitation (AR), aortic stenosis, aortic root dilation, and aortic dissection. The aetiology of BAV is notably intricate, involving a spectrum of genes and polymorphisms. Moreover, BAV lays the groundwork for an array of structural heart and aortic disorders, presenting varying degrees of severity. Establishing a tailored clinical approach amid this diverse range of BAV-related conditions is of utmost significance. In this comprehensive review, we delve into the epidemiology, aetiology, associated ailments, and clinical management of BAV, encompassing imaging to aortic surgery. Our exploration is guided by the perspectives of the aortic team, spanning six distinct guidelines. Methods: We conducted an exhaustive search across databases like PubMed, Ovid, Scopus, and Embase to extract relevant studies. Our review incorporates 84 references and integrates insights from six different guidelines to create a comprehensive clinical management section. Results: BAV presents complexities in its aetiology, with specific polymorphisms and gene disorders observed in groups with elevated BAV prevalence, contributing to increased susceptibility to other cardiovascular conditions. The altered hemodynamics inherent to BAV instigate adverse remodelling of the aorta and heart, thus fostering the development of epigenetically linked aortic and heart diseases. Employing TTE screening for first-degree relatives of BAV patients might be beneficial for disease tracking and enhancing clinical outcomes. While SAVR is the primary recommendation for indicated AVR in BAV, TAVR might be an option for certain patients endorsed by adept aortic teams. In addition, proficient teams can perform aortic valve repair for AR cases. Aortic surgery necessitates personalized evaluation, accounting for genetic makeup and risk factors. While the standard aortic replacement threshold stands at 55 mm, it may be tailored to 50 mm or even 45 mm based on patient-specific considerations. Conclusion: This review reiterates the significance of considering the multifactorial nature of BAV as well as the need for further research to be carried out in the field.

## 1. Introduction

The bicuspid aortic valve (BAV) is characterized by the presence of two commissures instead of the usual three [1]. BAV is the most common congenital heart disease (CHD), affecting approximately 1–2% of the general population [2]. The exact cause of BAV disease remains unclear, although it has been associated with various genetic syndromes and disorders such as Shone complex, Kabuki syndrome, and Marfan syndrome, as well as genetic variations and mutations [3,4]. Initially, BAV was thought to be a connective tissue disease like Marfan syndrome; however, subsequent observational and clinical studies have shown that BAV is not as extensively involved with connective tissue as Marfan syndrome [5,6]. Nevertheless, BAV patients still have a higher risk of aortic complications compared to those with a tricuspid aortic valve. A significant proportion of BAV patients requiring aortic valve replacement (AVR) or repair surgery also require concomitant aortic surgery due to BAV-related aeropathy [7,8]. Despite the high rates of aortic surgery in BAV patients, there is ongoing debate regarding the threshold for concomitant aortic surgery based on aortic dimensions [9,10]. In this review, we examine the epidemiology and aetiology of BAV, the relationship between BAV and aortopathies, and the surgical management of BAV in accordance with the current guidelines from AATS, EACTS/ESC, and AHA/ACC.

## 2. Materials and Methods

A comprehensive review of the literature was conducted by searching multiple electronic databases, including PubMed, Ovid, Scopus, and Embase, to identify and gather pertinent studies. A total of 84 papers were utilized as references to thoroughly discuss the bicuspid aortic valve. To assess the clinical management of bicuspid aortic valve disease in accordance with guidelines, the following were utilized: ACC/AHA’s 2022 Aortic Disease guidelines and 2020 Valve Disease guidelines, along with ESC/EACTS’s 2021 Valve Disease guidelines and 2014 Aortic Disease guidelines. Furthermore, the 2018 AATS guideline was consulted for the evaluation of bicuspid aortopathy.

## 3. Epidemiology of Bicuspid Aortic Valve

**Figure 1:**
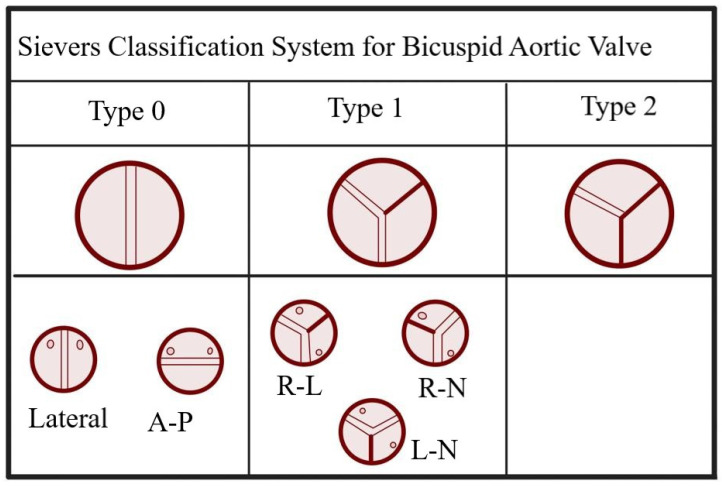
Sievers classification system for BAV. *A-P: Anteroposterior; R-L: Right-left coronary cusp fusion; R-N: Right-noncoronary cusp fusion; L-N: Left-noncoronary cusp fusion*.

BAV has a prevalence of 0.77–1.4% (potentially higher when asymptomatic patients are included) [11]. While BAV patients generally do not experience issues during infancy and childhood, they may develop various aortic valve abnormalities (such as stenosis and insufficiency) and encounter aortic problems later in life, including root dilatation, rupture, and dissection. Consequently, BAV represents the congenital heart disease (CHD) associated with the highest mortality rate [12]. BAV can be classified into three main groups: type-0, which consists of two equal cusps without a raphe; type-1, the most common group, characterized by the fusion of two cusps; and type-2, the rarest type, involving the fusion of three leaflets (Figure 1). Type-1 BAV accounts for 90–95% of cases and further subdivides into three subgroups: R-L (fusion of the right coronary cusp and the left coronary cusp), R-N (fusion between the right coronary and non-coronary cusps), and L-N (rarely seen, fusion between the left coronary and non-coronary cusps) [13]. The R-L subgroup, which is the most prevalent among the subgroups of Type-1 BAV, has been associated with neural crest cell migration issues [14]. Clinically, it exhibits a more favourable prognosis compared to the R-N subgroup, which is believed to be caused by an imperfection in the eNOS gene [15].

## 4. Aetiology of Bicuspid Aortic Valve

Despite being the most prevalent congenital heart disease, the aetiology and pathogenesis of bicuspid aortic valve morphology remain partially understood. However, the prevailing consensus within the literature suggests that this morphological condition is underpinned by a robust and intricate genetic basis.

### 4.1. Bicuspid Aortic Valve and Genetic Background

The occurrence of BAV within families is notably 5 to 15 times more prevalent than in the general population, pointing towards a likely genetic basis for this correlation [16]. Importantly, the male-to-female ratio in cases of BAV is evenly distributed at 1:1, which contrasts with the gender patterns observed in numerous acquired heart conditions [16]. In a study by Boureau et al., in which they focused on patients with calcific aortic valve disease, it was revealed that isolated cases of calcific aortic valve disease were more likely to show tricuspid morphology. Conversely, cases of calcific aortic valve disease presenting in a familial pattern showed predominantly bicuspid valve morphology [17]. Emphasizing the criticality of the issue, Tessler et al. emphasized the importance of performing echocardiographic evaluation in first-degree relatives of individuals diagnosed with BAV [18].

Examining the congenital syndromes associated with BAV holds substantial significance in unravelling the formation and potential pathogenesis of BAV itself. As depicted in Table 1, the incidence of BAV within congenital heart diseases provides insightful data. For instance, a notable correlation exists between BAV and the Shone complex, a condition characterized by a defect in the myocardial structural protein (MYH6). Remarkably, approximately 9 out of 10 patients with the Shone complex exhibit BAV, which is a severe form of left heart structural abnormality. Additionally, BAV is present in about one-third of patients with ventricular septal defect (VSD), suggesting a possible link to neural crest cell migration processes. Particularly striking is the elevated prevalence of BAV in conditions such as Turner and Kabuki syndromes. These syndromes, known to disrupt valvular microenvironmental homeostasis due to genetic impairments, exhibit a prevalence of BAV exceeding 10 times that of the general population (21% vs. 2%). This recurrent presence of BAV in such syndromes underscores its intricate association with conditions involving compromised neural crest migration and gene abnormalities that potentially disrupt the valvular microenvironment [19,20,21,22,23,24,25,26,27,28,29,30,31].

Table 2 presents a comprehensive overview of the genes associated with bicuspid aortic valve disease, believed to contribute to the development of this specific valve morphology [32,33,34,35,36,37,38,39,40,41,42,43,44,45]. Upon closer examination of these genes and the pathways they participate in, alongside congenital heart syndromes commonly featuring BAV, the genetic foundation of BAV can be succinctly summarized as follows:

- Function and Dysfunction of Cardiogenesis-Polarization Genes: Notably, genes integral to cardiogenesis, such as GATA and NKX2-5, play a pivotal role. These genes are central to the establishment and proper functioning of the heart.
- Dysregulation of Genes Associated with Neural Crest Cell Migration: Genes like ROBO4, implicated in the regulation of neural crest cell migration, also feature in the genetic context of BAV. Dysfunction here might contribute to anomalies in cardiac development.
- Defects and Disorders in Genes Governing Valve Microenvironment Maintenance: The integrity of the valve microenvironment relies on genes like TGFB2 and TBX. Irregularities in these genes can potentially lead to disruptions in the microenvironment, affecting valve development.
- Gene Aberrations in Structural Aspects of Connective Tissues: Structural issues concerning connective tissues are influenced by gene disorders, including FBN1 deficiency. These genetic irregularities can give rise to problems in the structural integrity of tissues that constitute the cardiovascular system.

In essence, the genetic underpinnings of BAV encompass a complex interplay of various genetic factors and pathways. The intricate dance between genes related to cardiogenesis, neural crest cell migration, valve microenvironment maintenance, and connective tissue structure collectively shapes the development of BAV. This holistic understanding underscores the multifaceted nature of the genetic basis behind BAV’s manifestation.

### 4.2. Genetical Background of Bicuspid Aortic Valve and Aorta

Genetic factors play an important role in the formation of BAV morphology as well as in the development of related aortic problems. Specifically, when genes responsible for structurally regulating connective tissue encounter disruptions or when genes essential for maintaining the valve microenvironment experience dysregulation, the result is weakening of the aortic structure [6,7,8,9,20]. While it is crucial to acknowledge that genetic anomalies are not the sole origin of aortic pathologies, they are acknowledged as a constituent aspect of the overall pathogenic process, shedding light on the complex interplay between genetic factors and the emergence of aortic complications.

### 4.3. Hemodynamic Features of Bicuspid Aortic Valve

Epigenetic factors have been suggested to contribute to aortic complications in patients with BAV. The non-linear blood flow across the BAV and its direction, which is influenced by the specific subtype category of BAV pathology, play a significant role [46]. The turbulent jet flow over the BAV increases wall shear stress on the valve and the associated areas of the ascending root and ascending aorta. Consequently, high wall shear stress leads to various epigenetic changes in smooth muscle cells, endothelial cells, and valvular interstitial cells. Increased wall shear stress triggers the expression of proinflammatory cytokines and proteins, potentially resulting in the thickening of valve cusps and degeneration of the aortic media. Studies by Rashad et al. have demonstrated the upregulation of pro-atherogenic factors (such as ICAM1 and E-selectin), pro-angiogenic factors (such as KFL2), and pro-vascular fibrotic factors (such as NOS) in response to high wall shear stress, as observed in patients with BAV [47].

The hemodynamic changes associated with BAV also contribute to the different types of aortic dilatation and aneurysm formation observed in BAV aortopathy. Arch dilatation is more common in patients with R-N cusp fusion, with jet streams directed toward the arch, whereas ascending aortic aneurysms are more prevalent in patients with L-R fusions, with jet streams directed toward the ascending aorta [48]. In a study by Charitos et al. involving 361 BAV patients and 448 patients undergoing tricuspid AVR, no difference in aortic dilatation or an increase in the size of the middle root was observed between BAV and tricuspid AVR patients after surgery. The authors concluded that aortic dilatation in BAV is primarily due to valve hemodynamics rather than genetic factors [49]. Fungi et al. reported a study involving 431 patients who underwent either isolated AVR, AVR with ascending aorta replacement, or aortic root replacement between 1993 and 2019. Their findings indicated that concomitant aortic surgery during aortic valve surgery for BAV does not impact survival in patients with BAV whose ascending aorta diameter ranges from 40 mm to 45 mm [50].

## 5. Aortic Pathologies and Bicuspid Aortic Valve

### 5.1. Calcific Aortic Valve Disease

The prevalence of aortic stenosis in patients with BAV is higher compared to patients with tricuspid aortic valve (TAV), ranging from 21% to 53% [51,52]. BAV patients are particularly susceptible to aortic stenosis due to a combination of genetic and epigenetic factors influenced by altered hemodynamics [53,54]. Calcific aortic valve disease (CAVD) occurs because of remodelling processes within the valve microenvironment triggered by increased mechanical stress and genetic predisposition. Under heightened mechanical stress, valvular interstitial cells acquire osteoblastic properties and express proteins that contribute to mineralization, such as osteopenia and osteonectin. Additionally, avascularization genes are downregulated, leading to further mineralization. Furthermore, these cells secrete MMP9, which degrades the organic collagen matrix, ultimately resulting in ossification. CAVD is a progressive disease that can also impact the structure of the aortic root [52].

### 5.2. Aortic Regurgitation

Aortic regurgitation (AR) is frequently observed in patients with a BAV [55]. This can be attributed to the predisposition of BAV patients to develop calcific valve calcification, resulting in an asymmetrical and distorted anatomical valve structure, as demonstrated by ex vivo models [56,57]. Moreover, BAV patients with AR are at an increased risk of aortic dissection due to a combination of abnormal stress rheology, high stroke volume, and a thin, degenerated aortic wall [8].

### 5.3. Aortic Dilation

Aortic dilatation is frequently observed in a range of 20% to 84% of patients with BAV, primarily attributed to accelerated degeneration of the aortic media within the BAV microenvironment [54]. This degeneration occurs due to increased matrix metalloproteinase (MMP) activation, decreased fibrillin-1 (FBN1) expression, and elevated proinflammatory cytokine expression, influenced by both genetic and hemodynamic factors [58,59,60]. Notably, aortic dilatation progresses rapidly in BAV patients. Davies et al. examined aortic aneurysm status in patients with BAV and TAV, revealing that BAV patients are typically younger (49.2 vs. 64 years) and exhibit smaller aneurysm size (4.6 cm vs. 4.9 cm); however, the expansion rate is higher compared to patients with TAV [61]. Aneurysmal aortic dilatation in BAV patients exhibits variable morphology, with distinct features in terms of location and type, necessitating individual evaluation [20].

**Figure 2:**
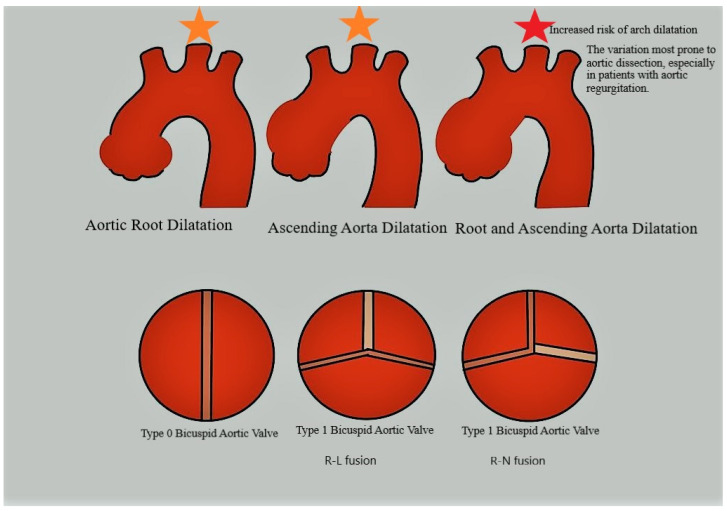
Aortic pathologies associated with BAV. Different coloured stars. Each valve morphology is associated with a specific aortic dilatation and changes in the risk of dissection. Type 0 is associated with root dilatation; type 1 R-L leaflet fusion is associated with ascending aorta dilatation; and type 1 R-N leaflet fusion is associated with root and ascending dilatation and an increased risk of dissection.

Among patients with BAV, sinus Valsalva and root dilatation are more prevalent in those with fusion of the left and right coronary cusps (Figure 2). Conversely, the more critical issue of aortic arch dilatation is more common in patients with fusion of the right coronary non-coronary cusp (Figure 2). Additionally, aortopathy varies depending on the type of valve dysfunction. Aggressive growth rate and root dilatation are more prominent in patients with aortic regurgitation, while asymmetric dilation at the tubular junction is more common in patients with stenosis [20].

### 5.4. Aortic Dissection

Aortic dissection is a potential complication in patients with BAV and associated aortopathy. The incidence of BAV-related aortic dissection can be as low as 0.6%, as reported by Wilson-Smith et al. [62]. However, the risk of aortic dissection in BAV patients is eight times higher compared to the general population [63]. Furthermore, aortic dissections tend to occur at younger ages in patients with BAV. It is recommended that patients with high-risk profiles, such as those with aortic regurgitation, a family history of dissection, a root phenotype, or a high rate of aortic diameter growth, should be offered prophylactic aortic replacement [64,65]. BAV patients who experience dissection are typically younger and may have lower blood pressure, but histopathological examination of resected aortas has revealed more severe medial degeneration [65]. Therefore, it is crucial to individualize the assessment of aortic dissection risk and the indications for prophylactic aortic replacement in patients with BAV disease.

## 6. Bicuspid Aortic Valve Clinical Management in Current Aortic Guidelines

The management of BAV presents a complex and critical challenge in cardiovascular medicine. Numerous factors must be taken into account, including BAV subtypes, valve dysfunction profile, patient symptoms, aortic root and arch size, growth rate, presence of hypertension, genetic profile, and family history of dissection. The watch-and-wait strategy is generally recommended only for asymptomatic patients with preserved left ventricular ejection fraction (LVEF) and normal aortic diameters, although such cases represent a small proportion of BAV patients encountered in clinical practice [7]. Medical treatment options for BAV patients are limited, with more than half requiring AVR during their lifetime, and 25% undergoing aortic replacement surgery [7]. Therefore, it is crucial to provide comprehensive counselling to patients with BAV disease and to establish appropriate surgical plans for this patient population and their families [20].

### 6.1. Familial Screening Recommendations

The prevalence of BAV among first-degree relatives of individuals with BAV stands at approximately 10–15%. Given the hemodynamic implications and genetically influenced dilation often linked to BAV, timely identification and routine monitoring of this condition hold paramount importance. In light of this, both American and European guidelines released over the past decade advocate for screening through transoesophageal echocardiography (TEE) among primary family relatives, with varying levels of evidence supporting this recommendation falling within class IIa/b [20,66,67,68,69]. Furthermore, research into genes associated with bicuspid aortic valve and their altered expression due to disrupted hemodynamics holds promise for the future development of a blood test-based algorithm. However, further investigation is required to advance this endeavour [67] (Table 3).

### 6.2. Surgical Management of Bicuspid Aortic Valve

Although guidelines [20,67,68,69] do not classify BAV as an independent indication for valve surgery and do not recommend AVR without symptoms or evident LVEF depression, certain studies yield mixed findings on the potential benefits of this treatment for seemingly asymptomatic BAV patients. Kang et al.’s research demonstrated enhanced survival among patients with aortic stenosis, a majority of whom had BAV and were initially categorized as asymptomatic prior to surgery [70]. However, in another study, the investigators did not observe a corresponding advantage from early surgery for BAV patients afflicted by aortic regurgitation, a condition affecting over 50 per cent of patients [71]. Hence, determining the optimal timing for surgery in cases involving BAV remains a subject of contention. For patients diagnosed with bicuspid aortic valve disease and an indication for AVR, current guidelines recommend surgical AVR (SAVR) with a class I recommendation, provided the patient is suitable for the procedure [68,69]. However, ongoing research is shedding light on whether this surgical intervention will involve repair or replacement in cases of aortic regurgitation. A meta-analysis published in 2019, comparing replacement and repair in aortic regurgitation, revealed that the perioperative outcomes of repair were comparable to those of replacement [72]. Notably, in the context of the bicuspid valve, a 2016 meta-analysis emphasized the potential feasibility and optimism associated with repair as a viable option [73]. In the European and American guidelines, it has been suggested that aortic valve repair in AR can be performed in accordance with the multidisciplinary team decision of experienced surgeons in experienced aortic centres, as a class IIb, level of evidence C recommendation [68,69].

### 6.3. Transcatheter and Rapid Deployment Valves for Bicuspid Aortic Valve

The indication for SAVR is clear for patients opting for aortic valve replacement [66,67,68,69], but alternatives that entail less risk are emerging for elderly or high-risk individuals who are unable to undergo SAVR independently [68]. Transcatheter AVR (TAVR) stands as a robust option in high- and intermediate-risk patient cohorts, yet considerable challenges arise within the context of BAV patients [74]. These challenges encompass anatomical intricacies, escalated annulus and cusp calcification, and the issue of coronary eccentricity. The latter’s significance lies in the potential to hinder access to necessary coronary pathways, particularly in the medium- and long-term [75]. Unlike surgical valves, aligning commissures and coronary access poses greater difficulty with transcatheter valves [76]. The issues of coronary access that are commonly observed in first-generation TAVR devices have been significantly mitigated through advancements in the design of next-generation valves and the establishment of standardized implantation techniques [76,77]. Nonetheless, there remains a shortage of conclusive evidence regarding coronary and commissural alignment, as well as coronary access following TAVR in cases involving bicuspid valves. However, studies centred around bicuspid valves available in the literature suggest that TAVR can be effectively and safely performed with a quite low risk of coronary occlusion in such valve types [78,79,80]. Moreover, TAVR might serve as a viable alternative for individuals with bicuspid valves who are deemed unsuitable candidates for traditional surgical approaches [78,79,80]. Notably, the 2020 ACC/AHA Guideline for the Management of Patients with Valvular Heart Disease [68] designates TAVR as a class IIb recommendation, level of evidence B, under specific conditions for bicuspid patients. The introduction of rapid deployment valves is geared towards streamlining surgical procedures and mitigating potential risks, offering a prospective substitute to traditional valve options [81,82]. Despite its limited scope, the literature displays promise in the utilization of rapid deployment valves for patients with bicuspid aortic valves. Efforts have been ongoing to refine techniques and address challenges identified in initial studies [82]. However, it is evident that comprehensive, well-designed investigations supported by robust evidence are imperative to attain a more comprehensive understanding of this matter (Table 4).

### 6.4. Management of Bicuspid Aortic Valve-Related Aorthopathy

Owing to a confluence of genetic, epigenetic, and hemodynamic factors, the prevalence of aortic dilatation in individuals with BAV spans a spectrum of 20–80%. Furthermore, the progression of this aortic dilatation often outpaces that observed in patients with TAV. This underscores the critical nature of managing aortic dilatation in the context of BAV. Initial considerations regarding surgical intervention in cases of BAV with aortic dilatation were influenced by thresholds established for conditions like Marfan syndrome [83,84]. However, with the accumulation of growing evidence over time and the adoption of a more tailored treatment approach, the criteria for aortic surgery in BAV patients vary based on specific circumstances. In broad terms, aortic replacement is strongly recommended as a Class I intervention when the aortic diameter reaches or exceeds 55 mm [20,66,67,68,69]. Nonetheless, both American and European guidelines exhibit a flexible approach when determining the threshold for surgical replacement or repair in cases involving BAV-associated aortopathy [20,66,67,68,69]. This adaptability is predicated on a diverse range of considerations, often categorized as class IIa or II b recommendations, supported by varying levels of evidence. These considerations encompass factors such as the morphology of the BAV, the severity of valve dysfunction (i.e., AR), concomitant aortic conditions (i.e., coarctation), the progression rate of aortic dilation, the stress placed on the weakened aortic wall, and a history of familial aortic dissection [20,66,67,68,69]. Furthermore, cases where simultaneous valve replacement is needed or specific genetic disorders (like ACTA2, Loeys–Dietz, Turner, or Marfan syndromes) are present can lead to a lowering of this threshold, often reaching 45 mm or even less [66,67,68,69,83,84]. The existence of distinct indication thresholds for various scenarios within BAV aortopathy highlights the significance of individualized assessment and strategic planning in addressing this intricate condition. Furthermore, a notable point underscored across all guidelines is the imperative for aortic interventions in BAV patients to be carried out with the collaboration of a multidisciplinary aortic team within specialized aortic centres, known for their comprehensive expertise and multidisciplinary approach (Table 5).

## 7. Conclusions

BAV’s intricate genetic foundation and disrupted hemodynamics are closely linked to a spectrum of aortic and cardiac disorders. Swift diagnosis, vigilant monitoring, and timely interventions hold paramount importance in BAV’s clinical management. To this end, the TTE screening of first-degree relatives of BAV patients is recommended. Furthermore, the identification of genetic and epigenetic hallmarks associated with BAV may pave the way for future hemogram-based risk assessments. Navigating interventions for BAV-associated issues remains an evolving field with limited and varied early intervention benefits. Further research is imperative. In BAV patients warranting AVR, SAVR emerges as the primary recommendation. However, the multidisciplinary aortic team can discern TAVR’s appropriateness for select cases. Notably, skilled surgeons, in collaboration with the multidisciplinary aortic team, can contemplate aortic valve repair for aortic regurgitation patients when suitable. The decision for aortic surgery hinges on multifaceted scenarios, underscoring its need for precision. In summation, BAV’s prevalent yet intricate nature calls for an individualized clinical approach. Its diverse manifestations demand a thorough patient-centred strategy.

## Figures and Tables

**Table 1:** Bicuspid aortic valve prevalence in congenital heart and vascular diseases.

Syndrome Name	BAV Prevalence *	Reference
Shone complex	88%	[19]
Ventricular septal defect (VSD)	30%	[20]
Turner syndrome	21%	[21]
Kabuki syndrome	21%	[22]
Adams–Oliver syndrome	13.3%	[23]
Hypoplastic left heart syndrome	11%	[24]
Tetralogy of Fallot	6.5%	[25]
Loesy–Dietz syndrome	6%	[26]
Marfan syndrome	4.7%	[27]
ACTA2 mutated patients	3%	[20]
Velocardiofacial syndrome	10%	[28]

* Bicuspid aortic valve prevalence in general is 0.5–2%.

**Table 2:** Genes Associated with Bicuspid Aortic Valve.

Gene Name	Variations	Reference
ROBO4	exon 13(c.2056+1G>T), R64C, A95T, T232M H411G, R568X, R64C, V247A, Y280S, G534Efs49, N622H, A749L, N510V, Ser327Pro	[32,39,41]
GATA	GATA4(rs6601627, S337G) GATA5(L233P, S19Y, Y143H, G166S, Y16D, T252P, Q3R, c.830C>T/p. P277L, p. (Gln3Arg)) GATA6(E38X)	[33,34,35,36,37,38,39,40,41]
NOTCH1	9q34-35, A1343V, P1390T, H1505del R1108x, T596M, P1797H, R1350L, P1377S,.873C>G/p. Tyr291	[39,42]
FBN1	-	[39]
SMAD6	C484F, P415L, K242NfsX300, Gly166VfsX23, G26_S27del, Y279X, Y288X, V239M, P257L, G271W, G406C, H408Q, R443H	[39]
TGFBR2	V387M	[35]
KCNJ2	R67W	[45]
NKX2-5	K192X	[39]
NRF2F	C96X	[39]
MCTP2	L847F, T545M	[39]
AXIN1/2	R841Q, A684V	[39]
NFATC1	P77L, V210M	[39]
TBX5	S372L; V263M	[39]
KFL13	Glu144-mutant	[43]
CELSR1	-	[44]

**Table 3:** American and European Guidelines on Screening for Patients with Bicuspid Aortic Valve.

Familial Screening	The American Association for Thoracic Surgery Consensus Guidelines on Bicuspid Aortic Valve–Related Aortopathy [20]	2014 ESC Guidelines on the Diagnosis and Treatment of Aortic Diseases [66]	2022 ACC/AHA Guideline for the Diagnosis and Management of Aortic Disease [67]	2020 ACC/AHA Guideline for the Management of Patients with Valvular Heart Disease [68]	2021 ESC/EACTS Guideline for the Management of Valvular Heart Disease [69]
Class I		-	-	-	-
Class IIa/b	Recommended to screen first-degree relatives of patients with BAV using echocardiography. (Class IIa, level of evidence B)	Recommended to screen first-degree relatives of patients with BAV using transthoracic echocardiography (Class IIa, level of evidence C).	Recommended to screen first-degree relatives of patients with BAV using transthoracic echocardiography (Class IIa, level of evidence B)	Recommended to screen first-degree relatives of patients with BAV using transthoracic echocardiography (Class IIb, level of evidence B).	Recommended to screen first-degree relatives of patients with BAV using transthoracic echocardiography. *

* The recommendation level was not specified.

**Table 4:** American and European Guidelines on Valvar Disease in Individuals with Bicuspid Aortic Valve.

	2020 ACC/AHA Guideline for the Management of Patients with Valvular Heart Disease [68]	2021 ESC/EACTS Guidelines for the Management of Valvular Heart Disease [69]
Class I	❖ For individuals indicated aortic valve replacement (AVR), surgical aortic valve replacement (SAVR) is advised if the patient is deemed suitable for the procedure following a personalized assessment of surgical feasibility.	*
Class IIa/b	❖ For individuals presenting with both BAV and symptomatic, severe aortic stenosis (AS), the option of transcatheter aortic valve replacement (TAVR) can be evaluated as an alternative to surgical aortic valve replacement (SAVR). This evaluation should encompass an assessment of the patient’s unique procedural risks, personal values, potential compromises, and preferences. Additionally, this alternative should be explored in cases where the procedure is carried out at a Comprehensive Valve Centre. (Class IIb, level of evidence B) ❖ For individuals diagnosed with both BAV and severe aortic regurgitation (AR) who fulfil the criteria for aortic valve replacement (AVR), the possibility of (surgical) aortic valve repair could be contemplated for specific patients, provided that the procedure is undertaken at a Comprehensive Valve Centre. (Class IIb, level of evidence C)	❖ Contemplation of surgical aortic valve repair is appropriate for certain patients, particularly when performed at experienced centres and anticipated to yield lasting outcomes. (Class IIb, level of evidence C)

* Although the ESC/EACTS guidelines do not provide an explicit recommendation, they acknowledge that surgical aortic valve replacement (SAVR) is typically more suitable in cases of bicuspid aortic stenosis (AS).

**Table 5:** American and European Guidelines on Aortic Repair for Patients with Bicuspid Aortic Valve.

Aortic Surgery	The American Association for Thoracic Surgery Consensus Guidelines on Bicuspid Aortic Valve–Related Aortopathy [20]	2014 ESC Guidelines on the Diagnosis and Treatment of Aortic Diseases [66]	2022 ACC/AHA Guideline for the Diagnosis and Management of Aortic Disease [67]	2020 ACC/AHA Guideline for the Management of Patients with Valvular Heart Disease [68]	2021 ESC/EACTS Guideline for the Management of Valvular Heart Disease [69]
Class I	❖ For individuals across all categories, an ascending aorta diameter exceeding 55 mm is indicated (Class I). ❖ For individuals across all categories, an aortic arch diameter exceeding 55 mm is indicated (Class I).	❖ For patients with a bicuspid valve, an ascending aorta diameter exceeding 55 mm is indicated (Class Ia, Level of Evidence C). ❖ For patients with a bicuspid valve and risk factors, a threshold of *50 mm* is recommended (Class Ia, Level of Evidence C). ❖ For patients with a BAV undergoing surgical aortic valve repair or replacement and having an aortic root or ascending aorta diameter of ≥45 mm, it is reasonable to consider concurrent replacement of the aortic root, ascending aorta, or both. This recommendation is particularly applicable when performed by experienced surgeons within a Multidisciplinary Aortic Team. (Class Ia, Level of Evidence C).	❖ For individuals across all categories, an ascending aorta nor root diameter exceeding 55 mm is indicated. (Class I, Level of Evidence B)	❖ For individuals across all categories, an ascending aorta nor root diameter exceeding 55 mm is indicated. (Class I, Level of Evidence B)	
Class II A/B	❖ For patients with a bicuspid valve and risk factors, a threshold of 50 mm is recommended (Class IIa, Level of Evidence B). ❖ Concomitant repair of the ascending aorta/root should be performed when the aortic diameter is ≥45 mm in patients undergoing cardiac surgery, with a Class IIa recommendation and Level of Evidence B. ❖ Concomitant repair of the aortic arch should be performed in patients undergoing cardiac surgery with an aortic arch diameter of ≥50 mm, with a Class IIa recommendation and Level of Evidence C. ❖ Repair of the ascending aorta/root may be performed in patients with an aortic diameter of ≥50 mm when the patients are at low surgical risk and operated on by an experienced aortic team in a centre with established surgical results, with a Class IIa recommendation and Level of Evidence C. ❖ Concomitant repair of the aortic arch may be performed in patients undergoing cardiac surgery with an aortic arch diameter of ≥45 mm, provided the patients are at low surgical risk and operated on by an experienced aortic team with established surgical results, with a Class IIb recommendation and Level of Evidence C.		❖ For patients with a bicuspid valve and risk factors, a threshold of 50 mm is recommended. (Class IIa, Level of Evidence B). ❖ For patients with a Bicuspid Aortic Valve (BAV) and a ratio of aortic size to their height of 10 cm^2^/m or higher, it might be advisable to contemplate surgery for replacing the aortic root, ascending aorta, or both. This suggestion is especially important when performed by skilled surgeons within a Multidisciplinary Aortic Team. (Class IIa, Level of Evidence B). ❖ For patients with a BAV undergoing surgical aortic valve repair or replacement and having an aortic root or ascending aorta diameter of ≥45 mm, it is reasonable to consider concurrent replacement of the aortic root, ascending aorta, or both. This recommendation is particularly applicable when performed by experienced surgeons within a Multidisciplinary Aortic Team. (Class IIa, Level of Evidence B) ❖ Among patients with a BAV, aortic size between 50 mm and 54 mm, low surgical risk, and no other risk factors, surgery may be considered. This is particularly the case when the procedure is carried out by experienced surgeons within a Multidisciplinary Aortic Team (Class IIb, Level of Evidence C).	❖ For patients with a bicuspid valve and risk factors, a threshold of 50 mm is recommended. (Class IIa, Level of Evidence B). ❖ For patients with a BAV who require SAVR and have an aortic sinuses or ascending aorta diameter of ≥45 mm, it may be reasonable to consider replacing the aortic sinuses and/or ascending aorta if the surgery is conducted at a Comprehensive Valve Centre (Class IIa, Level of Evidence B) ❖ Among patients with a BAV, aortic size between 50 mm and 54 mm, low surgical risk, and no other risk factors, surgery may be considered. This is particularly the case when the procedure is carried out by experienced surgeons within a Multidisciplinary Aortic Team (Class IIb, Level of Evidence B). ❖ For individuals with a BAV who fulfil the criteria for aortic sinus replacement, the option of valve-sparing surgery could be contemplated if the procedure takes place at a Comprehensive Valve Centre (Class IIb, Level of Evidence C)	❖ For individuals across all categories, an ascending aorta nor root diameter exceeding 55 mm is indicated. (Class II, Level of Evidence C) ❖ For patients with a bicuspid valve and risk factors, a threshold of 50 mm is recommended. (Class IIa, Level of Evidence C). ❖ For patients undergoing surgical aortic valve repair or replacement and having an aortic root or ascending aorta diameter of ≥45 mm, it is reasonable to consider concurrent replacement of the aortic root, ascending aorta, or both. This recommendation is particularly applicable when performed by experienced surgeons within a Multidisciplinary Aortic Team. (Class IIa, Level of Evidence c)

## Data Availability

All the data are available from the corresponding author.
